# Tracking dynamic changes in implementation strategies over time within a hybrid type 2 trial of an electronic patient-reported oncology symptom and needs monitoring program

**DOI:** 10.3389/frhs.2022.983217

**Published:** 2022-11-01

**Authors:** Justin D. Smith, James L. Merle, Kimberly A. Webster, September Cahue, Frank J. Penedo, Sofia F. Garcia

**Affiliations:** ^1^Department of Population Health Sciences, Spencer Fox Eccles School of Medicine, University of Utah, Salt Lake City, UT, United States; ^2^Department of Medical Social Sciences, Northwestern University Feinberg School of Medicine, Chicago, IL, United States; ^3^Departments of Psychology and Medicine, University of Miami, Coral Gables, FL, United States; ^4^Sylvester Comprehensive Cancer Center, Miller School of Medicine, University of Miami, Miami, FL, United States; ^5^Robert H. Lurie Comprehensive Cancer Center, Northwestern University, Chicago, IL, United States

**Keywords:** implementation strategies, modifications, adaptations, cancer symptom screening, tracking system

## Abstract

**Background:**

Longitudinal tracking of implementation strategies is critical in accurately reporting when and why they are used, for promoting rigor and reproducibility in implementation research, and could facilitate generalizable knowledge if similar methods are used across research projects. This article focuses on tracking dynamic changes in the use of implementation strategies over time within a hybrid type 2 effectiveness-implementation trial of an evidence-based electronic patient-reported oncology symptom assessment for cancer patient-reported outcomes in a single large healthcare system.

**Methods:**

The Longitudinal Implementation Strategies Tracking System (LISTS), a timeline follow-back procedure for documenting strategy use and modifications, was applied to the multiyear study. The research team used observation, study records, and reports from implementers to complete LISTS in an electronic data entry system. Types of modifications and reasons were categorized. Determinants associated with each strategy were collected as a justification for strategy use and a potential explanation for strategy modifications.

**Results:**

Thirty-four discrete implementation strategies were used and at least one strategy was used from each of the nine strategy categories from the Expert Recommendations for Implementing Change (ERIC) taxonomy. Most of the strategies were introduced, used, and continued or discontinued according to a prospective implementation plan. Relatedly, a small number of strategies were introduced, the majority unplanned, because of the changing healthcare landscape, or to address an emergent barrier. Despite changing implementation context, there were relatively few modifications to the way strategies were enacted, such as a change in the actor, action, or dose. Few differences were noted between the trial's three regional units under investigation.

**Conclusion:**

This study occurred within the ambulatory oncology clinics of a large, academic medical center and was supported by the Quality team of the health system to ensure greater uptake, uniformity, and implementation within established practice change processes. The centralized nature of the implementation likely contributed to the relatively low proportion of modified strategies and the high degree of uniformity across regions. These results demonstrate the potential of LISTS in gathering the level of data needed to understand the impact of the many implementation strategies used to support adoption and delivery of a multilevel innovation.

**Clinical trial registration:**

https://clinicaltrials.gov/ct2/show/NCT04014751, identifier: NCT04014751.

## Introduction

Due to advances in screening and treatment, the 5-year survival rate upon a cancer diagnosis is close to 70%, and there are almost 17 million cancer survivors in the US (1). Despite advances in early detection and treatments that extends survivor longevity, survival benefit is often offset by chronic and debilitating cancer- and treatment-related symptoms that compromise health related quality of life (2). Cancer patients experience disruptive physical and psychosocial symptoms that are often under-addressed. Research indicates that one in five cancer survivors experience uncontrolled pain (3), and around 32% meet Diagnostic and Statistical Manual of Mental Disorders criteria for a mental health diagnosis (e.g., adjustment, anxiety, sleep, mood) (2, 4). Therefore, providing optimal cancer care requires systematic symptom monitoring (5).

Tools that capture patient-reported outcomes (PROs) are emerging as a way to bridge the gap between patient experiences and clinician understanding (6). In oncology, PROs are assessed by engaging patients on their physical and psychological symptoms, functioning, quality of life, and supportive care needs. Incorporating PROs into routine oncology practice has been shown to improve patient outcomes, care satisfaction, and quality of life (7, 8). However, most studies evaluating programs to monitor and manage patient-reported outcomes (PROs) *via* electronic health records (EHRs) have been limited to efficacy trials and not implemented within routine practice of large healthcare systems (9).

Despite available guidance on integrating PROs as a standard of care (10), additional strategies are needed to promote their consistent and sustained implementation (11–13). Tracking and reporting implementation strategies is critical to determining under what circumstances they achieve their effects (14) and for promoting rigor and reproducibility in implementation research. Moreover, reporting and tracking of implementation modifications can be used to demonstrate fidelity to the strategies per the study protocol or, conversely, track and assess protocol deviations. Strategies are often adapted, modified, and discontinued based on several multilevel factors, such as emerging barriers and facilitators and evidence of low effectiveness. Therefore, it is crucial to capture and track these modifications within implementation studies (15).

Systems for tracking implementation strategy use and modification over time have been developed (16–20). However, among the limitations to existing tracking methods are: (1) they lack specificity in accordance with strategy reporting standards; (2) they largely collect data retrospectively or with wide time spans during the study rather than routinely throughout the implementation process; (3) the majority have been developed or applied *post-hoc* and relied on existing data sources that might have lacked the necessary detail on the strategy and how it was enacted.

To improve upon existing tracking systems, and fill gaps in the current literature, Smith and colleagues developed the Longitudinal Implementation Strategies Tracking System (LISTS), a robust, dynamic tool for measuring, monitoring, reporting, and guiding strategy use and modifications (21–23). LISTS was iteratively developed within the National Cancer Institute's Improving the Management of symPtoms during And following Cancer Treatment (IMPACT) research consortium—a Cancer Moonshot^SM^ program. The primary aim of LISTS is to track implementation strategies by capturing detailed data in near-real time on strategy use and modification that can be readily combined, synthesized, and compared within and between implementation projects. Secondarily, the system was developed to allow for tailoring strategies, assessing effectiveness, and evaluating costs of implementation strategies. LISTS was designed in alignment with (a) implementation strategy reporting and specification standards (14), (b) the Expert Recommendations for Implementing Change (ERIC) taxonomy (24), and (c) the Framework for Reporting Adaptations and Modifications to Evidence-based Implementation Strategies (FRAME-IS) (15). Use of LISTS over the course of 15 months in three randomized effectiveness-implementation hybrid trials (21–23) indicated that LISTS was feasible, usable, and led to meaningful data on strategy use and modification.

This study sought to demonstrate the capability of LISTS in tracking the use and modification of strategies to support implementation of the cancer patient-reported outcomes (“cPRO”) system across oncology care practices in a large healthcare system. cPRO consists of the Patient Reported Outcomes Measurement Information System (PROMIS^®^) computer adaptive tests (CATs) (25, 26) of (1) Depression (PROMIS Item Bank v1.0-Depression); (2) Anxiety (PROMIS Item Bank v1.0-Anxiety); (3) Fatigue (PROMIS Item Bank-Fatigue v1.0); (4) Pain Interference (PROMIS Item Bank v1.1-Pain Interference); and (5) Physical Function (PROMIS Item Bank v1.1-Physical Function), along with two supportive care checklist items (covering psychosocial and nutritional needs). Cancer center patients are asked to complete an assessment before each medical oncology visit (but no more than once a month). We report here on (a) the strategies used to support cPRO implementation, (b) the most common implementation strategy modifications made, which strategies and strategy types were modified, and, (c) which modifications were planned or unplanned, and the reasons for modifications. Additionally, we use this study as a use case to demonstrate the utility of using LISTS to populate the Implementation Research Logic Model (IRLM) (27) when reporting the results of an implementation trial. The IRLM can provide a useful visual of the conceptual relationships between determinants of implementation, strategies, and targeted outcomes.

## Materials and methods

This study was approved by the Northwestern University Institutional Review Board (STU00207807).

### Setting and participants

The study occurred at outpatient oncology settings across multiple hospitals that are part of the Northwestern Medicine healthcare system. Existing regional units (Central, North, and West) served as the clusters for a stepped wedge trial (28). In total, 32 clinical units participated across the three regions. All regions include medical centers/hospitals and specialty clinics for the diagnosis and management of cancer. The study population included any adult clinician (physician, nurse, social worker, dietician) administering cancer care at a medical oncology clinic; oncology clinic administrative staff; and eligible patients (confirmed cancer diagnosis and receiving oncology services within the past 12 months).

The participants involved in the completion of the LISTS tool in this study included a team comprised of one of the principal investigators (SFG), co-investigators (KAW, JDS), one of whom is an implementation scientist (JDS), and the project coordinator (SC). Implementers in the health system who enacted the implementation strategies were regularly consulted regarding LISTS data by members of the LISTS team *via* email, phone calls, and one-on-one conversations, but did not interact with the LISTS tool or the data entry system. This team has been involved and/or led implementation research studies and all members have familiarity with implementation science terms, theories, and concepts. However, only JDS has formal training in implementation science and thus guided the coding and classification of data elements. All team members contributed to the coding and agreed on the results reported.

### Study design and procedures

The overall study used a cluster randomized, modified stepped wedge design, using a type 2 hybrid effectiveness-implementation approach spanning 4 years (28). This approach allowed for the evaluation of both the cPRO effectiveness as well as the implementation outcomes associated with the implementation strategies. The design leveraged the healthcare system's three geographic and operational regions (Central, North, West) of 32 total clinical units. Regions were pseudo-randomly assigned to the roll-out sequence with 3-month steps. The Central region was the first cluster at the request of system leadership. West and North were then randomly assigned to the second and third spots in the sequence. For each regional cluster, a multicomponent “package” of implementation strategies was used to increase adoption and reach of cPRO. The package consisted primarily of strategies that were system-wide, which were introduced immediately prior to the crossover in the stepped wedge to evaluate their impact on implementation. cPRO usage data prior to the crossover provided an “implementation as usual” comparison.

### Longitudinal implementation strategies tracking system (LISTS)

#### Procedures and content

LISTS was used to track implementation strategy use and modifications. The LISTS team used observation, study records (meeting notes, calendars), and reports from implementers (*via* in-person, phone, and email inquiry) to document implementation strategy use, modifications, and discontinuations. When modification or discontinuation occurred, these were documented as planned or unplanned, reasons and person involved in the decision were recorded. To increase the accuracy of reporting, LISTS procedures involve the use of a timeline follow-back procedure (29) in which members of the research and implementation teams met every 3 months (quarterly) to complete LISTS, including entry of the data into a relational Research Electronic Data Capture (REDCap) (30) data entry system developed for LISTS. The team reported on strategy use and modifications at the study, region, and clinical unit levels as appropriate.

#### Data elements and capture

The data elements in LISTS were captured in REDCap, and the framework was drawn from multiple sources with widespread use and familiarity to the field of implementation. First, for strategy specification and reporting, we used the recommendations outlined by Proctor et al. (14). These elements include *naming* (using language consistent with the existing literature) and *defining* (operational definitions of the strategy and its discrete components) the strategy; specifying the *actor* (who enacts the strategy), *action* (active verb statements concerning the specific actions, steps, or processes), *action targets* (the strategy's intended target according to a conceptual model or theory), *temporality* (duration of use and interval or indication for use), *dose* (how long the strategy takes each time), and the *implementation outcome(s)* (implementation processes or outcomes likely to be affected).

Second, to assist LISTS users in naming strategies using language consistent with the existing literature, the tool is prepopulated with each of the 72 discrete strategies from the Expert Recommendations for Implementing Change (ERIC) compilation (24). The team completing LISTS used the ERIC compilation of strategies as a prompt and taxonomy for characterizing the strategies used. Detailed operational definitions were entered given the often vague nature of the ERIC strategy categories/types. Third, we used the Proctor et al. (31) taxonomy of implementation outcomes to provide users with agreed upon definitions for acceptability, adoption, appropriateness, cost, feasibility, fidelity, penetration/reach, and sustainability/sustainment. Fourth, LISTS included the complete list of determinants from the Consolidated Framework for Implementation Research (CFIR) (32) for users to select which determinant the strategy is hypothetically linked with, either as a barrier to overcome or a facilitator to be leveraged. This conceptual linking is consistent with the generalized theory of implementation research (27) and other theoretical and conceptual models used in the field (33–35), and will assist users in preparing the justification.

Finally, to capture the modifications made to the implementation strategies over time, we incorporated elements of the Framework for Reporting Adaptations and Modifications Expanded to Evidence-based Implementation Strategies (FRAME-IS) (15) for specific strategies, and additional elements related to project-level modifications. Consistent with the FRAME-IS, our data capture tool allows for updating already-entered strategies to indicate modifications to any aspect already described in this section and the discontinuation of a strategy. For both strategy modifications and discontinuation, branching logic prompts questions concerning the *reason* for a strategy change (e.g., ineffective, infeasible), *who* was involved in the strategy change decision (e.g., leadership, research team, clinicians), and whether the strategy change was *planned* (e.g., part of an a priori protocol) or *unplanned* (e.g., response to emergent implementation barrier). It is commonplace to add strategies during implementation for various reasons, which can be planned (e.g., as part of an adaptive or optimization study design) or unplanned. Unique to LISTS, when a strategy is added, the same “was it planned or unplanned” and “who was involved” questions are prompted along with the reason with response options of “to address an emergent barrier” or “to complement/supplement other strategies to increase effectiveness.” When a new strategy is added, the data elements for reporting and specifying as described above are also prompted. The full LISTS codebook with each data entry field as well as REDCap coding syntax are available in the primary LISTS paper (23). Most germane to the current study, we adhered to the definitions of CFIR constructs provided at https://cfirguide.org/constructs/ and used Additional File 6 (https://static-content.springer.com/esm/art%3A10.1186%2Fs13012-015-0209-1/MediaObjects/13012_2015_209_MOESM6_ESM.docx) from Powell et al. (24) for implementation strategy definitions and codes.

#### Timeframe of strategy reporting

Use of LISTS in this study began January 21, 2020. The study start date (official project period start date) was September 1, 2018 and start of implementation in the first region in the cluster randomized stepped-wedge sequence was December 23, 2019. While LISTS reporting began well into the project, reporting of previously used and currently in use strategies was comprehensive and included strategies prior to start of the project period that were instrumental to obtaining grant support for the study. These were conceptualized as part of the implementation preparation phase, defined as occurring prior to implementation of the innovation (i.e., cPRO) (36). Meetings related to LISTS occurred approximately quarterly through May 26, 2022, at which time data was pulled to conduct the current analysis.

### LISTS data display and output

**Figure 1:**
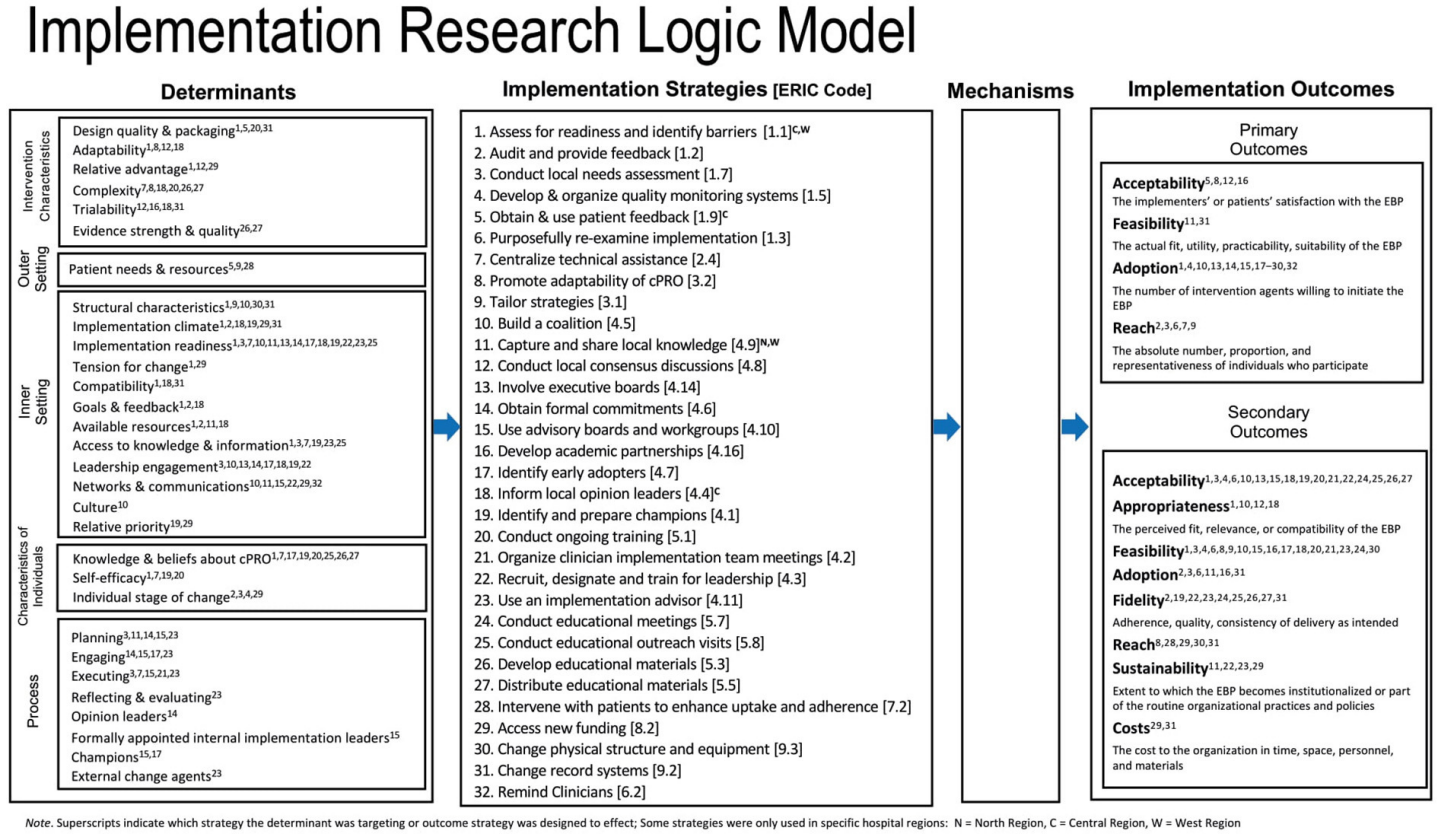
Timeline of implementation strategy use and modifications by phase. The number on each bar represent the associated ERIC strategy that can be found in Additional File 6 of Powell et al. (24).

To aid in visualizing and interpreting the complex relationships between the data elements captured in the LISTS tracker, a notated timeline (Figure 1) was created that spans the length of the study to date. We also utilized the Implementation Research Logic Model (IRLM) (27) to aid readers by organizing the relationships between implementation determinants, strategies, and their purported primary and secondary outcomes. This also allowed us to critically appraise the utility of LISTS data output by assessing its fit with a tool that helps specify and synthesize implementation projects with rigor. This step could inform further refinements to the type of data captured by the LISTS methodology.

## Results

### Implementation strategies used

A total of 34 discrete implementation strategies were documented as having been used between January 2015 and May 2022. While the formal trial described here began September 1, 2018, the team decided to capture strategies used during preparation for the trial, which included pilot studies and strategies that made submission of the grant application possible (e.g., partnership formation with the healthcare system). These strategies were coded into the ERIC categories (37) and all nine were represented. The category with the most strategies (*n* = 13) were from “develop stakeholder interrelationships,” followed by “use evaluative and iterative strategies” (*n* = 8) and “train and educate stakeholders” (*n* = 5). Only one strategy was used from each of “provide interactive assistance,” “support clinicians,” “utilize financial strategies,” and “engage consumers.” The remainder were from “change infrastructure” (*n* = 2) and “adapt and tailor to context” (*n* = 2). Most strategies (*n* = 28) were prospective (i.e., planned to be used *a priori* as part of the study protocol) and were used across all three regions of the healthcare system (*n* = 29). Research staff (*n* = 28) and/or quality improvement leaders (*n* = 27) served as the primary actor of the strategy (totals are not exclusive to one actor or the other). Figure 1 presents a timeline and key dates (study start), phases (preparation and implementation), color-coded strategy categorizations, and notation if the strategy was only used in one or two regions of the healthcare system. Detailed strategy definitions and their associated ERIC codes are available in Additional File 6 (https://static-content.springer.com/esm/art%3A10.1186%2Fs13012-015-0209-1/MediaObjects/13012_2015_209_MOESM6_ESM.docx) from Powell et al. (24).

### Implementation strategy modifications

Modifications to strategies can be categorized into two types. First, the introduction and discontinuation of a strategy (i.e., use) constitutes a protocol-level modification. That is, the study protocol is modified concerning which strategies are used and when. Second, modifications can occur to the way a strategy is enacted. That is, a change to one of the specifications of a strategy: actor, action, action target, temporality, dose, or outcomes/barriers addressed. The majority of modifications in this study were protocol modifications in which strategies were either introduced or discontinued per an a priori implementation plan. By extension, the majority of discontinuations to strategies were planned as opposed to unplanned. However, six strategies were unplanned introductions during the implementation phase to either to augment another strategy to increase effectiveness (*n* = 4) or to address an emergent barrier (*n* = 2). Relatively few (*n* = 6) of the strategies that were used involved a modification to the strategy specification. Action (*n* = 3) and dose (*n* = 3) were the most common specifications modified, followed by the action target (*n* = 2) and actor (*n* = 1). Two strategies involved multiple specification modifications. Notations are provided in Figure 1 for unplanned stoppages and introductions, and for those that had modifications to their specification during the study. Finally, the individuals involved in making the decision to modify the strategies were also coded, and they included the research team (*n* = 2 strategies), program leaders and administrators (*n* = 2 strategies), clinicians and healthcare staff (*n* = 2 strategies), implementers and trainers (*n* = 2 strategies), and patients (*n* = 1 strategy).

### Barriers and implementation outcomes targeted by strategies

**Figure 2:**
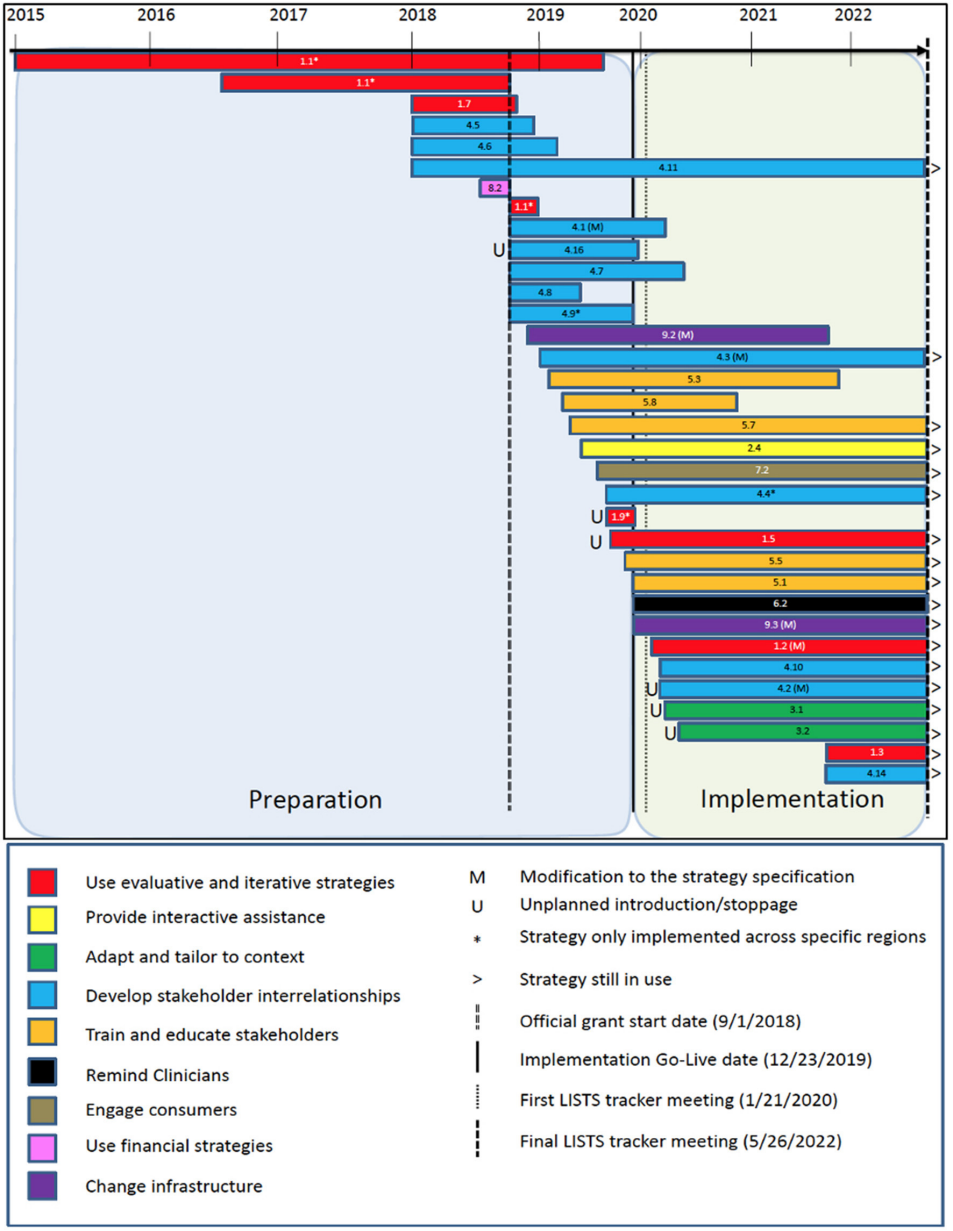
Implementation Research Logic Model (IRLM) populated with barriers, strategies, and outcomes. The Mechanisms field of the IRLM is left blank intentionally as that element is not captured within the LISTS method in its current version.

Implemented strategies targeted barriers across all five CFIR domains. Most strategies were used to overcome barriers in the inner setting (*n* = 26, 37%), followed by intervention characteristics (*n* = 17, 25%), individuals (*n* = 14, 20%), process (*n* = 9, 12%), and outer setting (*n* = 4, 6%). Strategies could target multiple determinants. Figure 2 provides further detail regarding the CFIR determinants coded by strategy.

Strategies were used primarily to increase adoption (*n* = 23, 68%), followed by reach (*n* = 5, 15%), acceptability (*n* = 4, 12%), and feasibility (*n* = 2, 6%) related to cPRO implementation. Regarding secondary outcomes, most strategies targeted feasibility (*n* = 19, 58%), followed by acceptability (*n* = 18, 55%) and fidelity (*n* = 9, 27%). Costs (*n* = 1, 6%) was the least targeted secondary outcome. A single primary outcome was selected and multiple secondary outcomes could be selected. Figure 2 presents a direct population of the IRLM using data from LISTS with superscripts to indicate the proposed barriers and outcomes associated with each strategy per best practice. Hospital region differences (i.e., Central, West, North) are also specified *via* notation in Figure 2.

## Discussion

Tracking the use and modification of implementation strategies is critical to ensure the rigor and reproducibility of implementation research (14). Despite the centrality of strategies in this scientific field, far too little attention has been paid to accurate reporting of strategies and how they change over time at the protocol and specification levels (23, 38). LISTS was developed to more accurately capture the dynamic nature of implementation strategy use and modifications over time in implementation research. Using a timeline follow-back procedure, strategies are evaluated on a routine basis at relatively short intervals (every 1–3 months) to capture and document modifications. This study is a demonstration of the utility of LISTS for strategy use and modifications that occurred over a 4-year-long cluster randomized stepped wedge trial using a type 2 effectiveness-implementation hybrid approach of cancer patient-reported outcomes (cPRO) symptom monitoring in a large urban and suburban healthcare system. The results demonstrate the potential of LISTS in gathering the type and granularity of data needed to understand the impact of strategies in implementation studies of complex, multilevel innovations.

Results indicated that 34 discrete implementation strategies were used, and at least one strategy was included from each of the nine strategy categories from the ERIC taxonomy. Since partnerships are crucial for implementation (39, 40), it was unsurprising that the category with the most strategies was “develop stakeholder interrelationships” (*n* = 12), and “evaluative and iterative strategies” was second (*n* = 7). Given the scope and complexity of this strategic implementation effort to effect system-wide change, the need for multilevel strategies to cut across ERIC categories seems reasonable and necessary. However, there is limited literature to contextualize this finding, specifically whether it is consistent with other implementation efforts. A study of opioid risk management implementation in the Department of Veterans Affairs found that project sites used an average of 23 strategies and a range of 16–31 discrete strategies. The most used strategies came from the ERIC categories of “adapt and tailor to the context,” “develop stakeholder interrelationships,” and “evaluative and iterative strategies,” which is consistent with our results. Adaptations to cPRO (*n* = 2) were few in comparison, perhaps because it is an electronic screener and simpler compared to the opioid risk management intervention.

Concerning the implementation strategy protocol, it was not surprising to see that most of the strategies used (28 of 34) were planned and relatively few modifications occurred to the strategies themselves once in use, which included no unplanned discontinuations, only six unplanned strategy introductions, and six unplanned modifications to a strategy's specification. The nature of the healthcare system and the experience of the study team are likely important determinants to consider when interpreting these results. This study occurred within the ambulatory oncology clinics of a large, academic medical center. As such, implementation was centralized, supported by established practice change processes, and championed by the Quality team of the health system to ensure greater uptake and uniformity across regions and clinics. This gave investigators considerable control over the protocol. Concerning the study team, there was a high degree of prior knowledge and experience related to PRO implementation in this specific healthcare system (9, 25, 26, 41). Relatedly, this study represented an attempt to improve and expand on the implementation of an already-in-use innovation (i.e., PROs), allowing for the specification of planned, targeted, strategic initiatives informed by prior data on identified barriers and effective facilitators. As such, there was a high degree of confidence in the protocol as designed. We believe these contextual factors contributed to fewer modifications.

It is worth noting that this study began prior to the COVID-19 pandemic, which had profound effects on healthcare delivery (42). Of the six strategies characterized as unplanned additions of specification modification, three were added during March and April, 2020 in direct response to the challenges associated with in-clinic cPRO assessment caused by COVID-19. Specifically, a clinician support team was organized to provide protected time to reflect on the implementation effort, share lessons learned, and determine needed supports. Despite the challenges, the centralized nature of the implementation seems to have counterbalanced the effects of COVID-19 mitigation measures on clinic operations. Two other unplanned additions occurred in September 2019, shortly before the intervention start date, were to augment other strategies to increase effectiveness. These included multimethod efforts to monitor data systems to check cPRO use quantitatively, and a patient advisory council to gather patient feedback regarding cPRO implementation. Though unplanned, these strategies served to provide a feedback loop to evaluate the ongoing implementation of cPRO. Later in implementation (September 2021), due to feedback from patients and data indicating that completion rates were lower than expected, the cPRO assessment was changed from a computer adaptive test version to a fixed-length version (called “cPRO Short”) to reduce the administration time with the goal of increasing patient response rates.

Concerning strategy use and modifications and the study design, it was important to carefully track and demonstrate that the implementation was consistent across the three regions of the Northwestern healthcare system, which served as the clusters in the stepped-wedge trial design. In most stepped-wedge designs, it is important to have the same implementation strategy across clusters for internal validity (43). However, it can be difficult to achieve this in implementation trials as strategies are often tailored to some extent to align with the contextual factors of the participating clinics or other units (44, 45). In this study, the contextual factors were relatively homogeneous across the regions and the centralized implementation support efforts further contributed to fewer region-level modifications to the protocol. Documenting the differences, or lack thereof, across study clusters aids with interpretation of the results. In this study, we can be confident that regional differences are not attributable to the implementation strategy package (given consistency in the strategies used across regions), but to other factors should they differ. Had there been meaningful variation in strategies across the regions, careful documentation of that variation would help the researchers' interpretation of differences in the findings by region.

The number of strategies that began during implementation preparation (*n* = 24) was two and a half times the number of strategies that began at or after implementation (*n* = 10). Consistent with what one might expect, “evaluative and iterative strategies,” such as “assessing for readiness and identifying barriers and facilitators” and “developing and implementing tools for quality monitoring” began years before implementation began and even before the grant period. Similarly, strategies within the “develop stakeholder interrelationships” (e.g., “Obtain formal commitments. “Promote network weaving,” “Inform local opinion leaders”) and a financial strategy of “Alter incentive/allowance structures” also began before the grant period. The remaining strategies (*n* = 26) began once funding was available through the grant. At the time the data were pulled for this analysis (May 16, 2022), 16 strategies were still being used to support cPRO implementation.

This study demonstrates that LISTS can be used to track strategy use and modifications at the protocol and specification levels; however, there are a number of considerations and potential future advancements to LISTS that could increase the utility and validity of the data. The use of the IRLM to visualize the data from LISTS concerning the relationships between strategies and the barriers and outcomes targeted provides useful information. The superscripts show that many implementation strategies were used to address prominent barriers. Barriers in the inner setting were most commonly the target of strategies used in this trial, with intervention characteristics being second most frequent and out setting determinants being the least frequent. More granular patterns of strategy-determinant relationships could be undertaken in a subsequent analysis of the data presented here. Similarly, implementation outcomes are conceptually connected to more than one, and in most cases many, strategies. Conceptually, each association is understandable and justifiable but the sheer number of relationships raises questions about the specificity of each strategy target and interpretation of effects that can be attributed a singular strategy. Although LISTS data can be used to populate the IRLM (Figure 2), further pruning and prioritization of the barriers and outcomes targeted might be needed to make it more useful and testable (e.g., causal path analysis). Additionally, the mechanisms that are part of a causal path analysis will need to be specified as LISTS in its current form does not prompt users to propose mechanistic targets. The IRLM was used in conjunction with LISTS data as it is becoming a popular method for reporting the results of implementation studies [see *articles in Special Supplement of the Journal of Acquired Immune Deficiency Syndrome*; (46)].

## Future directions

We envision LISTS being used in a variety of implementation studies with various research questions and designs. Tracking strategy with LISTS or similarly rigorous tools use will allow the field to advance our understanding of strategy effects. Comprehensive tracking of key elements of strategy use, specification, and modification could unlock the “black box” of what works when and under what contextual conditions. LISTS provides a uniform collection method to facilitate synthesis as the results of a single study or trial inevitably have limitations. LISTS would benefit from additional research and refinement in a number of areas to be maximally useful to the field. First, although LISTS captures details regarding which strategies were used and modified, and to some extent why, the current tool does not capture the efficiency or effectiveness of the strategy on outcomes. This aspect requires appropriate research designs such as optimization and factorial designs (47, 48). LISTS is tailor made to be the strategy collection method for such investigations. LISTS currently requires significant knowledge of implementation science models and frameworks, namely CFIR and ERIC in the context of this study, but also implementation theory to specify mechanisms for strategy selection. This represents a potential limitation to adoption and to use by implementation practitioners and community partners. Relatedly, it is yet to be determined the acceptability and utility of LISTS to implementers outside of the context of rigorous implementation research. Lastly, visual or graphical display of strategy use and modifications is also a potential area of future development for LISTS data. Figure 1 in this article provides one way to visually display the timeframe of strategy use with some notations for protocol and specification modifications. Such a figure is useful for portraying when and which type of strategy was introduced and discontinued but less detail can be included regarding strategy specification and why modifications occurred. Moreover, the figure does not capture different strategies, with meaningfully unique operationalizations, within the same ERIC code, which may be an area of future development. Finally, the current process of creating a visual display such as Figure 1 is manual. Automated visualizations that are customizable by users is a future direction for LISTS developers to consider. Despite the need for additional research on LISTS and potential refinements and additions, the LISTS method represents an advancement to other strategy tracking methods in the literature. Future research into the LISTS method should also formally examine the utility of the process and output from the perspective of the implementers and the research team.

## Conclusions

In conclusion, this study is the first to report implementation strategy use and modification over a multi-year period using LISTS, which was both feasible for use and resulted in meaningful and reliable data. While relatively few strategy modifications occurred within this study, due in large part to the centralized nature of the implementation support and the study being within one healthcare system, we demonstrated the potential utility of LISTS for capturing the type and granularity of data on modifications needed for rigor and reproducibility of implementation studies.

## Data availability statement

The raw data supporting the conclusions of this article will be made available by the authors, without undue reservation.

## Ethics statement

This study was approved by the Northwestern University Institutional Review Board (STU00207807). Written informed consent for participation was not required for this study in accordance with the national legislation and the institutional requirements.

## Author contributions

JS conceptualized the work reported in this article, wrote sections of the manuscript, provided critical edits to all manuscript sections, and provided consultation to the creation of figures. JM wrote sections of the manuscript, provided critical edits to all manuscript sections, analyzed the data, and created the figures. KW provided critical edits to all sections of the manuscript and made significant contribution to database organization. SC managed the database and reviewed the final version of the manuscript. SG and FP conceived of the overall trial and were awarded the grant supporting this work and provided critical edits to the manuscript. All authors contributed to and approved the manuscript to be published.

## Funding

Research reported in this publication was supported by grant R18 HS026170 from the Agency for Healthcare Research and Quality to SG and FP, by grant number UL1TR001422 from the National Institutes of Health's National Center for Advancing Translational Sciences, and the National Institute of Health training postdoctoral slot to JM (NLM; T15LM007124). The content is solely the responsibility of the authors and does not necessarily represent the official views of the National Institutes of Health.

## Conflict of interest

The authors declare that the research was conducted in the absence of any commercial or financial relationships that could be construed as a potential conflict of interest.

## Publisher's note

All claims expressed in this article are solely those of the authors and do not necessarily represent those of their affiliated organizations, or those of the publisher, the editors and the reviewers. Any product that may be evaluated in this article, or claim that may be made by its manufacturer, is not guaranteed or endorsed by the publisher.
